# Supplementary material for: Physician-Level Cost Control Measures and Regional Variation of Biosimilar Utilization in Germany
Source: Int J Environ Res Public Health. 2020 Jun 9;17(11):4113. doi: 10.3390/ijerph17114113 (PMC7313006; doi:10.3390/ijerph17114113)
Supplement: Supplementary file 1 [file ijerph-17-04113-s001.pdf]

Supplementary Material

Supplementary Table 1: Identification of drug agreements for biosimilar prescribing, web search and contact with physician associations.

Physician association	URL, last access 14.05.2018	Drug agreements (web search)	Date contacted	Drug agreements (material obtained and date responded)
Baden-Wuerttemberg	https://www.kvbawue.de/praxis/service/formulare-antraege-merkblaetter/	2013-2017	15.05.2018	04.06.2018: 2008-2012
Bavaria	https://www.kvb.de/verordnungen/arzneimittel/richtliniengesetze/	2013-2018	15.05.2018	18.06.2018: 2008-2011
Berlin	https://www.kvberlin.de/20praxis/60vertrag/10vertraege/arznei_und_heilmittel/index.html	complete	No inquiry	
Brandenburg	https://www.kvbb.de/praxis/verordnungen/arzneimittel/	2010-2018	15.05.2018	No response
Bremen	https://www.kvhb.de/arzneimittel-richtgr%C3%B6%C3%9Fen	2014-2018	15.05.2018	27.06.2018: 2008-2013
Hamburg	http://www.kvhh.net/kvhh/pages/index/p/177/0/g_id/428	2012-2017	15.05.2018	24.05.2018: 2008-2011
Hesse	https://www.kvhessen.de/fuer-unsere-mitglieder/recht-und-vertrag/land/arzneimittel-vereinbarung/	2012-2018	15.05.2018	No response
Mecklenburg-Western Pomerania	http://www.kvmv.info/aerzte/22/01_Arzneimittel/Regionale_Vereinbarungen/index.html	-	15.05.2018	No response
Lower Saxony	http://www.kvn.de/Ueber-uns/Amtliche-Bekanntmachungen/	Agreements available, but no biosimilar regulation	15.05.2018	01.06.2018
North Rhine	https://www.kvno.de/10praxis/40verordnungen/10arzneimittel/a_z/index.html	2011-2018	15.05.2018	15.05.2018: 2008-2010
Rhineland-Palatinate	https://www.kv-rlp.de/mitglieder/vertraege/arzneimittel/	2016-2018	15.05.2018	30.05.2018: 2008-2015
Saarland	https://www.kvsaarland.de/ vertraege-der-kvs	complete	No inquiry	
Saxony	https://www.kvs-sachsen.de/mitglieder/ vertraege/	2013-2018	15.05.2018	25.05.2018: 2008-2012
Saxony-Anhalt	https://www.kvsa.de/praxis/verordnungsmanagement/arzneimittel/arzneimittelvereinbarung.html	2018	15.05.2018	18.07.2018: 2008-2017
Schleswig-Holstein	https://www.kvsh.de/KVSH/index.php?StoryID=299&kat=7&search=	complete	No inquiry	
Thuringia	https://www.kv-thueringen.de/mitglieder/vertraege/a/arzneimittelvereinb/index.html	complete	No inquiry	

Westphalia-Lippe	https://www.kvwl.de/arzt/recht/kvwl/amv_hmv/index.htm	complete	No inquiry

Supplementary Table 2: Number of physician practices with biologic prescriptions 2013.

Physician association	ESA	Filgrastim	Somatropin
Schleswig-Holstein	378	127	58
Hamburg	194	73	38
Bremen	67	21	28
Lower Saxony	962	284	206
Westphalia-Lippe	823	208	173
North Rhine	938	196	161
Hesse	608	126	107
Rhineland-Palatinate	469	137	72
Baden-Wuerttemberg	1,471	569	275
Bavaria	1,507	522	261
Berlin	298	134	34
Saarland	147	31	17
Mecklenburg-Western Pomerania	295	107	50
Brandenburg	312	65	30
Saxony-Anhalt	385	133	27
Thuringia	279	125	20
Saxony	439	189	48
Total	9,572	3,047	1,605

Supplementary Table 3: Cost-control measures for biosimilars by physician associations, 2009-2015, ESA.

Physician association	2009	2010	2011	2012	2013	2014	2015
Bavaria	2	0	0	0	1	2	2
Bremen	0	0	0	1	1	2	2
Hesse*	0	0	0	2	2	2	0
Rhineland-Palatinate	2	2	2	2	2	2	2
Schleswig-Holstein	2	2	2	2	2	1	0
Baden-Wuerttemberg	0	0	0	0	0	0	0
Berlin	2	0	2	2	2	2	2
Brandenburg*	0	2	2	2	2	2	2
Lower Saxony	0	0	0	0	0	0	2
North Rhine	2	2	2	2	2	2	2
Saxony	0	2	2	0	0	0	0
Westphalia-Lippe	2	2	2	2	2	2	2
Hamburg	0	0	2	2	2	2	2
Saarland	0	2	2	2	2	2	2
Saxony-Anhalt	0	0	2	2	2	2	2
Thuringia	0	1	1	1	0	0	0
Mecklenburg-Western Pomerania*	0	0	0	0	0	0	0

* No complete data on cost-control measures available and therefore excluded from analysis, 0: no measure, 1: priority prescribing, 2: biosimilar quota.

Supplementary Table 4. Cost-control measures for biosimilars by physician associations, 2009-2015, filgrastim.

Physician association	2009	2010	2011	2012	2013	2014	2015
Bavaria	0	0	0	0	1	1	1
Bremen	0	0	0	1	1	2	2
Hesse*	0	0	0	0	2	2	0
Rhineland-Palatinate	0	0	0	0	0	1	1
Schleswig-Holstein	0	0	2	2	2	1	0
Baden-Wuerttemberg	0	0	0	0	0	0	0
Berlin	0	0	0	0	0	0	0
Brandenburg*	0	0	0	0	0	0	0
Lower Saxony	0	0	0	0	0	0	1
North Rhine	0	0	0	0	0	0	1
Saxony	0	0	0	0	0	0	0
Westphalia-Lippe	0	0	0	1	1	1	1
Hamburg	0	0	0	0	0	0	0
Saarland	0	0	0	0	0	0	1
Saxony-Anhalt	0	0	0	0	0	0	0
Thuringia	0	0	0	0	0	0	0
Mecklenburg-Western Pomerania*	0	0	0	0	0	0	0

* No complete data on cost-control measures available and therefore excluded from analysis, 0: no measure, 1: priority prescribing, 2: biosimilar quota.

Supplementary Table 5. Cost-control measures for biosimilars by physician associations, 2009-2015, somatropin.

Physician association	2009	2010	2011	2012	2013	2014	2015
Bavaria	0	0	0	0	1	1	2
Bremen	0	0	0	1	1	2	2
Hesse*	0	0	0	0	2	0	0
Rhineland-Palatinate	0	1	1	1	1	1	1
Schleswig-Holstein	0	0	0	0	0	1	0
Baden-Wuerttemberg	0	0	0	0	0	0	0
Berlin	0	0	0	0	0	0	0
Brandenburg*	0	0	0	0	0	0	0
Lower Saxony	0	0	0	0	0	0	1
North Rhine	0	2	0	0	0	0	1
Saxony	0	0	0	0	0	0	0
Westphalia-Lippe	0	0	0	1	1	2	2
Hamburg	0	0	0	0	0	0	0
Saarland	0	2	0	0	0	0	1
Saxony-Anhalt	0	0	0	0	0	0	0
Thuringia	0	1	0	0	0	0	0
Mecklenburg-Western Pomerania*	0	0	0	0	0	0	0

* No complete data on cost-control measures available and therefore excluded from analysis, 0: no measure, 1: priority prescribing, 2: biosimilar quota
